# Isolation and Immunomodulatory Effect of Homoisoflavones and Flavones from *Agave sisalana* Perrine ex Engelm.

**DOI:** 10.3390/molecules14051789

**Published:** 2009-05-12

**Authors:** Pi Yu Chen, Yuh Chi Kuo, Chin Hui Chen, Yueh Hsiung Kuo, Ching Kuo Lee

**Affiliations:** 1 Graduate Institute of Pharmacy, Taipei Medical University, 250 Wu Xin Street, Taipei 11031, Taiwan; E-mail: e023089103@tmu.edu.tw (P-Y.C), chinhui@mail.ypu.edu.tw (C-H.C.); 2 Institute of Life Science, Fu-Jen University; 510 Chung Cheng Rd, Hsinchuang, Taipei 24205, Taiwan; E-mail: 021553@mail.fju.edu.tw (Y-H.K); 3 Tsutzki Institute for Traditional Medicine, China Medical University, 91 Hsueh Shih Road, Taichung 40402, Taiwan; 4 Agricultural Biotechnology Research Center, Academia Sinica, 128, Sec. 2, Academia Road, Nankang, Taipei 115, Taiwan

**Keywords:** *Agave sisalana* Perrine ex Engelm., homoisoflavonoid, PBMC, IL-2, IFN-γ

## Abstract

Three known flavones and seven known homoisoflavonoids were isolated from the methanolic extract of the leaves of *Agave sisalana* Perrine ex Engelm. Their structures were elucidated on the basis of spectroscopic analysis. The isolated compounds were also evaluated for immunopharmacological activity. PBMC were used as target cells, and cell proliferation was determined by ^3^H-thymidine uptake. (±)-3,9-Dihydroeucomin (**4**), dihydrobonducellin (**5**), and 5,7-dihydroxy-3-(4′-hydroxybenzyl)-4-chromanone (**7**) showed inhibitory effects on PBMC proliferation activated by PHA with IC_50_ values 19.4, 73.8, and 58.8 µM, respectively. All three compounds significantly inhibited the production of IL-2 and IFN-γ in activated PBMC in a concentration-dependent manner.

## Introduction

The genus *Agave* belongs to the Amaryllidaceae family, with more than 300 species throughout the world. They occur natively in the arid and tropical regions of the Western Hemisphere, particularly Mexico, Central America, and South Taiwan. In Taiwan, species of the genus *Agave*, such as *A. americana* and *A. sisalana*, have been widely cultivated since 1918 in the southern parts for the fiber industry. Several *Agave spp*. have been used in the treatment of scabies, tumors, syphilis and dysentery, and as insecticides [1]. 

**Figure 1:**
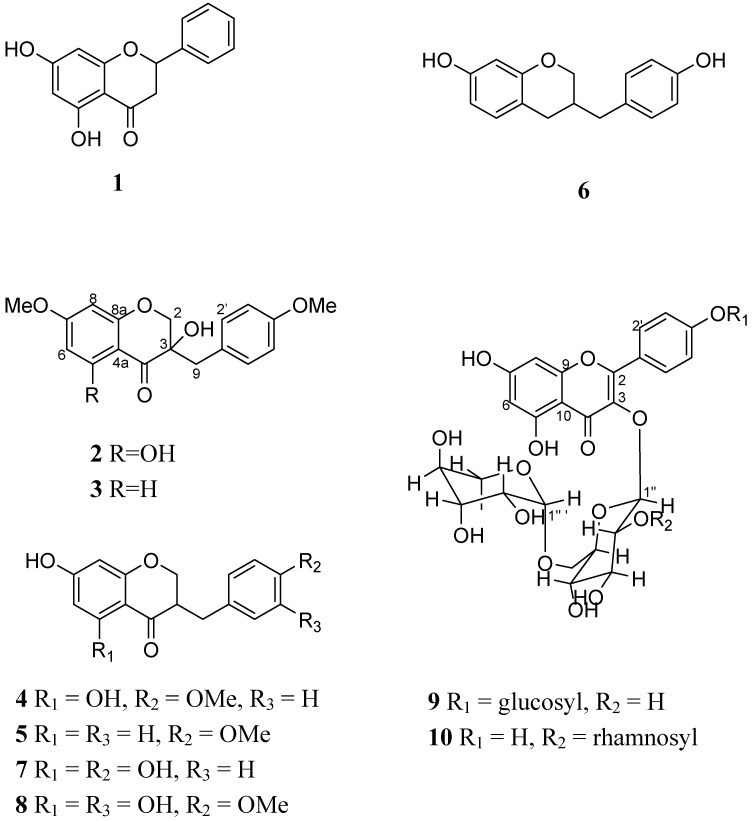
Chemical structure of compound **1-10**.

In recent years homoisoflavonoids have been reported that have the activities of cytotoxicity (MCF-7) [2] and inhibitory activity against the enzyme COX-2 [3]. Due to their structural similarities, homoisoflavonoids have been reported to display activity comparable to that of flavonoids, some of which show immunopharmacological activity [4]. The activation and clonal expansion of human peripheral blood mononuclear cells (PBMC) play important roles in the generation of immune responses. Antigens or phytohemagglutinin (PHA) can stimulate resting PBMC to proliferate and differentiate. It has been demonstrated in many previous studies that a series of cytokines such as interleukin-2 (IL-2) and interferon-γ (IFN-γ) are important in the growth of PBMC induced by antigens or PHA [5]. In this paper, three flavonoids: 5,7-dihydroxyflavanone (**1**) [6], kaempferol 3-rutinoside-4′-glucoside (**9**) [7], and kaempferol 3-(2^G^-rhamnosylrutinoside) (**10**) [8] and seven homoisoflavonoids: 7-*O*-methyleucomol (**2**) [9], 3′-deoxysappanone (**3**) [10], (±)-3,9-dihydroeucomin (**4**) [2], dihydro-bonducellin (**5**) [11], 7-hydroxy-3-(4-hydroxybenzyl)chromane (**6**) [12], 5,7-dihydroxy-3-(4′-hydroxy-benzyl)-4-chromanone (**7**) [2], and 5,7-dihydroxy-3-(3′-hydroxy-4′-methoxybenzyl)-4-chromanone (**8**) [13] (Figure 1) were isolated from methanolic extraction of *Agave sisalana* Perrine ex Engelm. We then used PBMC isolated from human peripheral blood as target cells to evaluate the immunomodulatory action of these compounds. 

## Results and Discussion

The compounds were separated by open column and HPLC. Although all of these compounds are known, they were all isolated for the first time from this plant. The ^13^C-NMR data of compound **9** was not reported in previous studies, so an assignment of all carbon signals of compound **9** is given in this paper.

**Figure 2:**
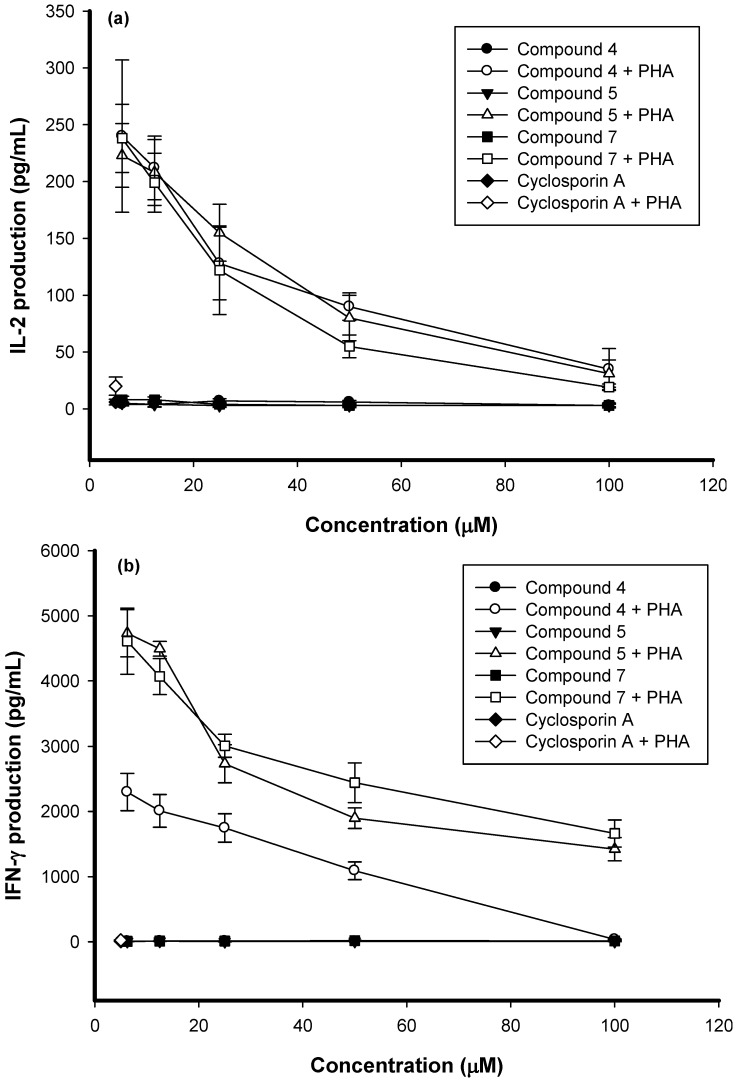
The impairment of IL-2 and IFN-γ production in PBMC treated with compounds **4**, **5**, and **7**. PBMC (2×10^5^ cell/well) were treated with 6.25, 12.5, 25, 50, and 100 µM of compounds 4, 5, and 7 with or without PHA (5 µg/mL) for 3 days. Cyclosporin A (5 µM) was used as positive control. Then, the cell supematants were collected and IL-2 **(a)** and IFN-γ **(b)** concentration were determined by EIA. Each of the point is the mean ± SD of three independent experiments.

All compounds were used to test the immunomodulatory action but only three of them displayed any such activity. The data indicated that vehicle DMSO (0.1%) did not affect cell proliferation in resting or PHA-stimulated PBMC. The IC_50_ values of (±)-3,9-dihydroeucomin (**4**), dihydrobonducellin (**5**), and 5,7-dihydroxy-3-(4′-hydroxybenzyl)-4-chromanone (**7**) on activated PBMC proliferation were 19.4, 73.8, and 58.8 µM, respectively. Compared with previously research [4], the IC_50_ values of these homoisoflavonoids showed potent flavonoid-like activities, especially (±)-3,9-dihydroeucomin (**4**). To determine whether the impairment of activated PBMC proliferation was related to cytokine production, the cell supernatants were collected and the production of IL-2 and IFN-γ assayed by enzyme immunoassays (EIA). The stimulated production of IL-2 and IFN-γ in activated PBMC was significantly suppressed by compounds **4**, **5**, and **7** in a dose-dependent manner (Figure 2). All three compounds had no significant cytotoxic effect on PBMC determined by trypan blue staining (data not shown). The inhibitory mechanisms may involve the impairments of IL-2 and IFN-γ production in PBMC. These results suggested that compounds **4**, **5**, and **7** had immunosuppressive effects.

(±)-3,9-Dihydroeucomin (**4**) showed better inhibition potency of PBMC proliferation. If the C-4′ position was occupied by a hydroxyl group, the activity became 3-fold lower than with a methoxyl group. Lacking a C-5 position hydroxyl group, compound **5** showed decreased inhibition activity. From these results it can be inferred that a C-4′ methoxyl group and C-5 hydroxy group seem to be necessary groups for the activity. A detailed SAR study and determination of the possible mechanism of action could be carried out in the future.

## Experimental 

### General

NMR spectra were recorded in CD_3_OD on a Bruker DRX-500 instrument. MS spectra were recorded on JEOL SX-102A Mass Spectrometer. For normal phase and reverse phase open column chromatography, Silica-gel (Merck, Geduran^®^ Si60 0.063-0.2 mm) and ODS gel (BioSil, ODS-W 45-55μ) was used. Semipreparative HPLC was performed on a HITACHI L-7110 instrument equipped with a Refractive Index detector (Thermo Separation Products) and a normal phase column (Phenomenex, Luna Silica column, 5 μm, 10×250 mm column, flow rate 3 mL min^-1^) or a reverse phase column (Merck, Lichrospher^®^ 100 RP-8 column, 10 μm, 10×250 mm, flow rate 3 mL min^-1^) were used to purified compounds.

### Plant material

The leaves of *A. sisalana* Perrine ex Engelm. (Amaryllidaceae) were collected in HengChun Town, PingTung county, Taiwan, in July, 2004. The material was identified by Dr. Chi I Chang, Science and Technology Graduate Institute of Biotechnology, National Pintung University. A voucher specimen (LCK9306) has been deposited in the Graduate Institute of Pharmacy, Taipei Medical University, Taipei, Taiwan.

### Extraction and isolation

The leaves of *Agave sisalana* Perrine ex Engelm. (61.1 kg) were cut into small pieces and extracted once with MeOH (100 L). The methanolic extract, after removal of the solvent under reduced pressure, was partitioned between ethyl acetate and deionized water. The ethyl acetate layer (69.6 g) was absorbed with silica gel (101.0 g) and then subjected to silica gel chromatography using a mixture of *n-*hexane-ethyl acetate-acetone-methanol to give fractions 1 to 8. Fraction 2 was purified by semipreparative normal-phase (NP) HPLC with an isocratic mixture of *n*-hexane-ethyl acetate (7:1) and than *n*-hexane-ethyl acetate-acetone (7:1:0.5) to afford compounds **1** (4.3 mg, *R*t 15.1 min), **2** (27.7 mg, *R*t 13.2 min), **3** (4.0 mg, *R*t 14.5 min), and **4** (15.3 mg, *R*t 18.9 min). Fraction 3 was purified by NP HPLC with an isocratic mixture of *n*-hexane-ethyl acetate (3.5:1) and then *n*-hexane-ethyl acetate- acetone (7:1:1) to obtain compounds **5** (58.8 mg, *R*t 18.8 min) and **6** (78.0 mg, *R*t 22.8 min). Fraction 4 was purified by NP HPLC with an isocratic mixture of *n*-hexane-ethyl acetate (3:1) to give compounds **7** (9.7 mg, *R*t 20.4 min) and **8** (2.1 mg, *R*t 22.0 min). The butanol layer (546.0 g) was separated by silica-gel column chromatography with chloroform-methanol to give ten fractions. Fraction 6 was separated by reverse phase column chromatography with isocratic solvent system of H_2_O-methanol- acetonitrile (2:1:1) to give compound **9** (149.8 mg). Compound **10** (41.8 mg, *R*t 18.9 min) was separated by semipreparative reverse-phase HPLC with an isocratic mixture of H_2_O-methanol-acetonitrile (78:11:11) from subfraction 7.

*NMR data for kaempferol 3-rutinoside-4′-glucoside* (**9**): ^13^C-NMR (CD_3_OD, 125 MHz) δ 179.5 (C, C-4), 166.2 (C, C-7), 163.0 (C, C-5), 161.1 (C, C-9), 158.8 (C, C-2), 158.6 (C, C-4′), 135.9 (C, C-3), 132.1 (CH, C-2′, 6′), 125.6 (C, C-1′), 117.1 (CH, C-3′, 5′), 105.8 (CH, C-10), 104.4 (CH, C-1′′), 102.3 (CH, C-1′′′), 101.6 (CH, C-1′′′′), 100.1 (CH, C-8), 95.0 (CH, C-6), 78.2 (CH, C-3′′), 78.1 (CH, C-5′′′′), 77.9 (CH, C-3′′′′), 77.2 (CH, C-5′′), 75.7 (CH, C-2′′), 74.9 (CH, C-2′′′′), 73.8 (CH, C-4′′′), 72.2 (CH, C-3′′′), 72.0 (CH, C-2′′′), 71.5 (CH, C-4′′), 71.3 (CH, C-4′′′′), 69.8 (CH, C-5′′′), 68.5 (CH_2_, C-6′′), 62.5 (CH_2_, C-6′′′′), 17.9 (CH_3_, C-6′′′); HRFAB MS (pos. ion mode) *m/z* 757.2211 (calcd for C_33_H_41_O_20_ 757.2191).

### Preparation of human peripheral blood mononuclear cells (PBMC)

PBMC were isolated from peripheral blood (20 mL) of healthy donors by the Ficoll-Hypaque gradient density method as described previously [14]. The PBMC layers were collected and resuspended to a concentration of 2×10^5^ cells/mL.

### Lymphoproliferation test

The lymphoproliferation test was modified from that previously described [14]. Various concentrations of compounds or the positive control cyclosporin A (6.25 µM) [4] were added to the cells and cultured for three days. After being pulsed with *^3^H*-thymidine (1 µCi/well; NEN) for 16 h, the cells were harvested on glass fiber filters and measure by a scintillation counting. The experiments for each compound were repeated three times and the data are indicated as mean ± SD.

### Determination of cytokine production in PBMC

The method was followed as previously described [4]. PBMC (2×10^5^ cell/well) were cultured with PHA alone or in combination with varying concentrations of compounds **4**, **5**, or **7** for 3 days. The cell supernatants were then collected and assayed for IL-2 and IFN-γ concentrations by enzyme immunoassays (EIA; R&D systems, Minneapolis, USA).

## Conclusions

Our results showed that homoisoflavones (±)-3,9-dihydroeucomin (**4**), dihydrobonducellin (**5**), and 5,7-dihydroxy-3-(4′-hydroxybenzyl)-4-chromanone (**7**), suppressed the production of IL-2 and IFN-γ. (±)-3,9-Dihydroeucomin (**4**) showed significant immunosuppressive effects. The saturated groups of C-4′ methoxyl and C-5 hydroxy seem to be necessary groups for the activity.
